# Role of *Streptococcus pneumoniae* extracellular glycosidases in immune evasion

**DOI:** 10.3389/fcimb.2023.1109449

**Published:** 2023-02-03

**Authors:** Bijina J. Mathew, Priyal Gupta, Tabassum Naaz, Rupal Rai, Sudheer Gupta, Sudipti Gupta, Shivendra K. Chaurasiya, Shashank Purwar, Debasis Biswas, Ashish Kumar Vyas, Anirudh K. Singh

**Affiliations:** ^1^ Department of Biological Science and Engineering, Maulana Azad National Institute of Technology, Bhopal, India; ^2^ Department of Microbiology, All India Institute of Medical Sciences, Bhopal, India; ^3^ Research and Development, 3B Blackbio Biotech India Ltd., Bhopal, India; ^4^ Abigail Wexner Research Institute, Nationwide Children’s Hospital, Columbus, OH, United States; ^5^ John C Martin Centre for Liver Research and Innovation, Liver Foundation Sonarpur, Kolkata, India; ^6^ School of Sciences, SAM Global University, Raisen, India

**Keywords:** *Streptococcus pneumoniae*, immune evasion, adherence, glycan, complement, biofilm, glycosidases, pneumococcus

## Abstract

*Streptococcus pneumoniae* (pneumococcus) typically colonizes the human upper airway asymptomatically but upon reaching other sites of the host body can cause an array of diseases such as pneumonia, bacteremia, otitis media, and meningitis. Be it colonization or progression to disease state, pneumococcus faces multiple challenges posed by host immunity ranging from complement mediated killing to inflammation driven recruitment of bactericidal cells for the containment of the pathogen. Pneumococcus has evolved several mechanisms to evade the host inflicted immune attack. The major pneumococcal virulence factor, the polysaccharide capsule helps protect the bacteria from complement mediated opsonophagocytic killing. Another important group of pneumococcal proteins which help bacteria to establish and thrive in the host environment is surface associated glycosidases. These enzymes can hydrolyze host glycans on glycoproteins, glycolipids, and glycosaminoglycans and consequently help bacteria acquire carbohydrates for growth. Many of these glycosidases directly or indirectly facilitate bacterial adherence and are known to modulate the function of host defense/immune proteins likely by removing glycans and thereby affecting their stability and/or function. Furthermore, these enzymes are known to contribute the formation of biofilms, the bacterial communities inherently resilient to antimicrobials and host immune attack. In this review, we summarize the role of these enzymes in host immune evasion.

## Introduction

*S. pneumoniae* (pneumococcus) is the major cause of community acquired pneumonia and the leading cause of preventable deaths in children under the age of five years (WHO; Bartlett and Mundy, 1995; Kyu et al., 2016; Le Roux and Zar, 2017; Angulo-Zamudio et al., 2019). The bacterium is also responsible for other significant diseases such as meningitis, bacteremia and otitis media (Obaro and Adegbola, 2002; Drijkoningen and Rohde, 2014). Pneumococcus typically colonizes the human nasopharynx asymptomatically. However, the bacterium can progress to different sites of the host body and cause aforesaid diseases. Although mechanisms involved in the transition of pneumococcus from asymptomatic colonization to a pathogenic state remain undefined, ability of the bacteria to survive the host immune response is central to the virulence and pathogenesis. A wide array of immune responses is activated by the host upon encounter with a foreign antigen/pathogen in order to clear the infection (Chaplin, 2010; Justiz Vaillant et al., 2022). The interaction of the bacteria with the host evokes an acute inflammatory response which involves the aggregation of phagocytic cells like neutrophils, platelets, and activation of the complement system. In order to persist, pneumococcus employs different mechanisms to evade such host immune responses. The polysaccharide capsule on the pneumococcal surface protects the bacteria from complement mediated killing and phagocytosis (Brown et al., 1983a; Brown et al., 1983b; Hyams et al., 2010). Furthermore, the capsule helps conceal surface ligands such as peptidoglycans, lipoteichoic acid, and other molecules to avoid activation of toll like receptor-mediated immune pathways (Skov Sorensen et al., 1988; Schwandner et al., 1999; Rajagopal and Walker, 2017). Pneumococcal surface associated glycosidases are another important group of molecules that contribute to pneumococcal virulence by facilitating nutrient acquisition, promoting adherence and impeding immune response. Pneumococcus encodes several surface associated glycosidases, including the major neuraminidase NanA, the β-galactosidase BgaA, and the N-acetylglucosaminidase StrH. These enzymes sequentially deglycosylate host glycans present on the cell surface or extracellular matrix as glycoproteins and/or glycolipids to release simple carbohydrates which are used as the carbon source by the bacteria (King et al., 2006). Furthermore, these enzymes contribute to adherence and immune evasion (King et al., 2004; King et al., 2006; Dalia et al., 2010; Blanchette et al., 2016). In this mini review, we summarize the role of pneumococcal surface glycosidases in subverting host immune response. Immune evasion mechanisms facilitated by these glycosidases fall into two broad categories; 1) defense, where these enzymes help bacteria achieve a physiological state which is resistant to host immune response, such as biofilms, and 2) offense, where these enzymes actively modify host immune effectors to render them ineffective or less effective such as deglycosylation of complement system proteins to compromise their stability/activity.

## Extracellular glycosidases in *S. pneumoniae*

**Figure 1:**
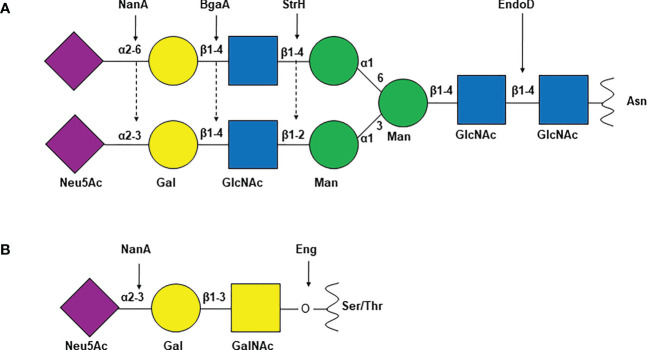
Common *S. pneumoniae* glycosidases and their glycan substrates. Schematic representation of **(A)** complex N-linked glycan and **(B)** core-1 O-linked glycan. Arrows indicate the target glyosidic bond of the enzyme above. NanA, neuraminidase A; BgaA, β-galactosidase A; StrH, N-acetylglucosaminidase StrH; EndoD, Endo-β-N-acetylglucosaminidase; Eng, O-glycosidase; Neu5Ac, N-acetylneuraminic acid or sialic acid; Gal, galactose; GlcNAc, N-acetylglucosamine; Man, mannose; GalNAc, N-acetylgalactosamine; Asn, asparagine; Ser, serine; Thr, threonine.

Different strains of *S. pneumoniae* encode anywhere from ten to twelve surface associated and/or secreted glycosidases which are known to modify host glycoconjugates including N and O-linked glycans, glycolipids, glycosaminoglycans, and other host derived carbohydrates (Berry et al., 1994; King et al., 2006; Marion et al., 2009). While most of these enzymes are covalently anchored to the cell wall peptidoglycan by the sortase A through the LPXTG motif, mechanisms of surface localization/secretion of the others are not well defined (**Table 1**). These enzymes include exoglycosidases which remove terminal sugars on a glycan structure and endoglycosidases which act on glycosidic bonds between sugar moieties that are not terminal. Different pneumococcal strains encode up to three neuraminidases NanA, NanB, and NanC. The major neuraminidase NanA is present in all the known pneumococcal genomes, however, NanB is present in 96% and NanC is encoded by only 51% of the isolates (Pettigrew et al., 2006). While the cleavage activity of NanB and NanC is limited to terminal α2-3 linked sialic acid, NanA is more promiscuous and can cleave α2-3 and α2-6 linked terminal sialic acid residues (Camara et al., 1994; Gut et al., 2008; Xu et al., 2008; Xu et al., 2011; Owen et al., 2015). Two β-galactosidases, BgaA and BgaC then remove galactose (Gal)β1-4 linked to N-acetylglucosamine (GlcNAc) or glucose and terminal Galβ1-3 linked to GlcNAc, respectively (Zahner and Hakenbeck, 2000; Jeong et al., 2009). Terminal GlcNAc residues that are β1-2 or β1-4 linked to mannose within complex N-linked glycans are removed by the action of N-acetylglucosaminidase StrH (Clarke et al., 1995; Pluvinage et al., 2011). Endo-β-N-acetylglucosaminidase, EndoD and O-glycosidase, Eng cleave chitobiose core of N-linked glycan and core -1 (Galβ1-3GalNAc) from O-linked glycans, respectively (Muramatsu et al., 2001; Caines et al., 2008) (**Figure 1**). Two α-fucosidases, SpGH29 and SpGH95 have distinct substrate specificities and, remove fucose from the various fucosylated glycans (Hobbs et al., 2019). Pneumococcus expresses other glycosidases which are involved in the hydrolysis of non-glycan carbohydrates such as glycogen and hyaluronic acid. The pullulanase SpuA hydrolyses bonds in glycogen and hyaluronate lyase Hyl breaks down hyaluronic acid (Berry et al., 1994; Li et al., 2000; Abbott et al., 2010).

**Table 1:** Extracellular glycosidases of *S. pneumoniae*.

Sl. No.	Glycosidase	Enzyme activity	CAZy Classification	Mechanism of surface localization	Substrate	Role in immune evasion	Reference
1	NanA	Neuraminidase	GH33	Anchored to surface through LPXTG motif	Siaα2-3Gal, Siaα2-6Gal, Siaα2-6GalNAc	Biofilm formation, deglycosylation of immune proteins	Camara et al., 1994; Xu et al., 2008; Parker et al., 2009;
2	NanB	Neuraminidase	GH33	Secreted, mechanism not defined	Siaα2-3Gal	May contribute to biofilm but has not been tested experimentally	Berry et al., 1996; Xu et al., 2008; Xu et al., 2011;
3	NanC	Neuraminidase	GH33	Secreted, mechanism not defined	Siaα2-3Gal	Not known	Xu et al., 2011; Owen et al., 2015;
4	BgaA	β-galactosidase	GH2	Anchored to surface through LPXTG motif	Galβ1-4GlcNAc, Galβ1-4Glu	Biofilm formation, deglycosylation of immune proteins	Zahner and Hakenbeck, 2000: Singh et al., 2014; Blanchette et al., 2016
5	BgaC	β-galactosidase	GH35	Surface anchored, mechanism not known	Galβ1-3GlcNAc	Not known	Jeong et al., 2009
6	StrH	*N*-acetylglucosaminidase	GH20	Anchored to surface through LPXTG motif	GlcNAcβ1-2Man, GlcNAcβ1-4Man, GlcNAcβ1-3Gal, GlcNAcβ1-6Gal	Not known	Clarke et al., 1995; Pluvinage et al., 2011;
7	EndoD	Endo-β-*N*-acetylglucosaminidase	GH85	Anchored to surface through LPXTG motif	GlcNAcβ1-4GlcNAc in Man3GlcNAc2 core of N-linked glycan	Not known	Muramatsu et al., 2001
8	Eng	*O*-glycosidase	GH101	Anchored to surface through LPXTG motif	Galβ1-3GalNAc from O-linked glycan	Not known	Marion et al., 2009
9	Hyl	Hyaluronate lyase	GH84	Anchored to surface through LPXTG motif	(GlcAβ1-3GlcNAc)β1-4(GlcAβ1-3GlcNAc) β1-4 linkage between disaccharide repeats	Not known	Li et al., 2000
10	SpuA	Pullulanase	GH13	Secreted, mechanism not defined	Glcα1-6Glc	Not known	Abbott et al., 2010
11	*Sp*GH29	α-fucosidase	GH29	Secreted, mechanism not defined	Fucα1-3 and Fucα1-4 linked in 3fucosyllactose and all four Lewis antigens (Lewis a, b, x, y)	Not known	Hobbs et al., 2019
12	*Sp*GH95	α-fucosidase	GH95	Anchored to surface through LPXTG motif	Fucα1-2 linked in 2fucocyllactose, H-antigen, Lewis b and y antigens	Not known	Hobbs et al., 2019

CAZy, Carbohydrate-Active enZYmes Database; GH, glycosyl hydrolase; Sia, sialic acid; Gal, galactose; GalNAc, N-acetyllactosamine; GlcNAc, N-acetylglucosamine; Man, mannose; Fuc, fucose; Glc, glucose.

Several of these enzymes contribute to pneumococcal colonization and virulence in a variety of ways (Dalia et al., 2010; King, 2010; Yamaguchi et al., 2020; Tseng et al., 2021). Pneumococcus utilizes carbohydrates as carbon and energy source. However, the upper and lower airway mucosal layers have a scarcity of free carbohydrates that can be utilized for pneumococcal growth. Surface associated glycosidases degrade complex glycans on extracellular matrix and cell surface glycoproteins, glycolipids, and glycosaminoglycans to release mono and/or dimeric carbohydrates which can be utilized by pneumococcus for growth. NanA, BgaA, StrH and EndoD can sequentially hydrolyze N-linked glycans on host proteins to support bacterial growth in the absence of glucose in chemically defined media (Burnaugh et al., 2008; Robb et al., 2017). O-glycosidase Eng has been shown to support the growth of pneumococci on O-linked glycans present on the human glycoprotein fetuin (Marion et al., 2009) and hyaluronate lyase Hyl, supports the growth on hyaluronic acid (Marion et al., 2012). NanA has been shown to contribute to adherence to the airway epithelia by removing terminal sialic acid residue to reveal cryptic receptors and by directly binding to human brain endothelial cells through a lectin-like domain present in the enzyme (Uchiyama et al., 2009). Additional neuraminidase NanB also plays role in colonization and sepsis (Manco et al., 2006). BgaA is shown to mediate pneumococcal adherence to host cells by binding to NanA exposed galactose (Gal)β1-4 linked on N-linked glycan and glycolipid through carbohydrate binding modules in the C-terminal region of the enzyme (Limoli et al., 2011; Singh et al., 2014). Glycans are critical components of glycoproteins and improper glycosylation of these proteins may lead to reduced stability and compromise their biological functions (Rudd et al., 2001; Sola and Griebenow, 2009). Pneumococcal surface glycosidases have been shown to remove glycans from a variety of host proteins involved in containing pneumococci and thereby reduce their pneumocidal effect (King et al., 2006; Dalia et al., 2010). The role of glycosidases in pneumococcal colonization has been elaborated in an elegantly written review by King (King, 2010).

## Glycosidases help build biofilms

A bacterial biofilm is a highly organized structure of surface attached communities encased in self-produced extracellular polymeric substances. Persistent infections by bacterial pathogens are largely associated with biofilm formation. *S. pneumoniae* colonization of the nasopharynx as well as diseases viz. otitis media and chronic rhinosinusitis are associated with biofilms (Hall-Stoodley et al., 2006; Sanderson et al., 2006; Hoa et al., 2009; Reid et al., 2009). Distinct biological and physiochemical characteristics of biofilms render the bacteria resistant to various antimicrobial agents and host immune responses (Chen and Wen, 2011; Bjarnsholt, 2013). The complement system is the first line of defense against *S. pneumoniae* and acts as an intermediate between innate and adaptive immune response (Merle et al., 2015). Activation of the complement cascade plays an important role in the recognition and clearance of pneumococci through phagocytosis and initiation of several inflammatory responses (Gil et al., 2022). This is evident from the observation that individuals with a deficiency in their complement are more prone to *S. pneumoniae* infection (Sjoholm et al., 2006; Skattum et al., 2011). An in-depth insight into the role of complement in pneumococcus biology is provided by Gil et al. (Gil et al., 2022).

Domenech et al., demonstrated that pneumococcal biofilms grown *in vitro* showed delayed complement activation due to the inhibition of the classical complement pathway as the deposition of C1q is significantly reduced on its surface (Domenech et al., 2013). They further demonstrated that *S. pneumoniae* cells in biofilms had reduced deposition of C3b, a central player in complement immunity, and were thereby less prone to opsonization (Domenech et al., 2013). Other than the classical pathway, the alternative pathway of complement immunity was also impaired in pneumococcal biofilms as indicated by the increased binding of its cascade down regulator factor H (Pathak et al., 2018). One of the pneumococcal surface proteins, PspC is involved in the augmentation of factor H, thus providing resistance to the pneumococcal biofilm by altering the complement amplification and subsequently avoiding recognition by C3b (Dave et al., 2001). The other important aspect of complement immunity is the clearance of pneumococci by phagocytosis using neutrophils, which was also impaired in pneumococcal biofilms (Domenech et al., 2013). It is to be noted that most of the evidence of biofilm associated resistance to complement mediated killing comes from studies using *in vitro* grown pneumococcal biofilms. Nonetheless, the role of complement in restricting otitis media, a biofilm associated disease makes a reasonable argument in favor of biofilm mediated protection against complement in this pneumococcal disease (Sabharwal et al., 2009). Furthermore, the persistence of pneumococci in the nasopharynx disease has been linked to the ability of the bacteria to form biofilm (Blanchette et al., 2016).

Pneumococcal glycosidases contribute to biofilm formation, thus helping in immune evasion likely by, 1) promoting adherence to the host surfaces, 2) facilitating nutrient acquisition, and 3) mediating bacterial cell aggregation. NanA plays an important role in the establishment of pneumococcal biofilm. A *nanA* deletion mutant of *S. pneumoniae* had a significant reduction in the biofilm formation ability when compared to the isogenic wild type strain (Parker et al., 2009). The role of NanA in biofilm was further confirmed by the inhibition of biofilm formation by small molecule inhibitors of the enzyme (Parker et al., 2009). While, NanA is known to contribute to the pneumococcal adherence to airway epithelia by exposing a cryptic receptor beneath sialic acid (Limoli et al., 2011) and by mediating direct binding to the brain endothelial cells (Uchiyama et al., 2009), this defect in biofilm formation was not attributed to the NanA mediated adherence (Parker et al., 2009). A layer of adherent *nanA* deletion mutant bacteria was observed suggesting that NanA contributes to biofilm formation by promoting the aggregation of planktonic pneumococci (Parker et al., 2009). NanA molecules from two different cells interact using either the two catalytic domains or one catalytic and one lectin domain leading to the formation of complex structures stabilized by intermolecular bonding (Sharapova et al., 2021).

Trappetti, et al. demonstrated that sialic acid promoted the growth of *in vitro* biofilms at the concentration normally found in saliva. Inoculation of sialic acid in the nasopharynx of the mouse significantly increased pneumococcal counts, further supporting the role of sialic acid in colonization and thereby likely in biofilm formation *in vivo* (Trappetti et al., 2009). This study eluded the role of a host derived carbohydrate, sialic acid in pneumococcal colonization and may have provided an early clue of the role of the pneumococcal neuraminidases in promoting *in vivo* biofilms by releasing sialic acid from the host sialylated glycoconjugates. Blanchette et al. reported that commonly available sugars like glucose, sucrose, and fructose are inhibitory to the biofilm formation whereas galactose, lactose, and sialic acid permit the growth of biofilm in mice nasopharynx further supporting the findings of Trappetti et al. They observed that *nanA* and *bgaA* mutant strains of *S. pneumoniae* failed to form biofilm in the nasal septa of mice (Blanchette et al., 2016). NanA and BgaA likely promoted biofilm formation by removing sialic acid and galactose, respectively, from the host glycans. While this study did not investigate the role of NanA and/or BgaA mediated adherence to the host epithelia in the formation of biofilm, this additional function of these enzymes may still contribute to the formation of the biofilms *in vivo*. Furthermore, it is possible that the other neuraminidase NanB may also contribute to biofilm formation *in vivo* by removing sialic acid from the host glycoconjugates. Although the direct role of NanB has not been investigated in the formation of biofilms, the observation that a *nanB* deletion mutant failed to proliferate in the airway of mouse is suggestive of its role in biofilm formation (Manco et al., 2006).

## Glycosidases modify immune proteins to escape the host immune response

### Modification of complement proteins

One of the major clearance mechanisms of *S. pneumoniae* by the host is opsonization *via* the complement system (King et al., 2004; Andre et al., 2017). C3b deposits on the bacterial surface which induces a neutrophil mediated phagocytosis. Most components of the complement system contain complex biantennary glycans (Ritchie et al., 2002). It has been shown that deglycosylation *via* exoglycosidases reduces the complement deposition on pneumococcus thus protecting it from opsonization (King et al., 2004; Singh et al., 2014). It is not clear exactly how the complement system is impaired leading to resistance against complement deposition. One of the possible mechanisms is that deglycosylation of the complement components directly reduces the complement deposition and another possibility is that the deglycosylation makes the complement components more susceptible to serum proteases which degrade them thus reducing the complement deposition on pneumococcus (King et al., 2004). Since the complement system is involved in both antibody dependent and antibody independent modes of clearance, it can be summarized that impairment of the complement system influences both the innate and adaptive immunity of the host.

### Modification of lactoferrin

Lactoferrin is a glycoprotein present on the mucosal membrane of the human airway. Lactoferrin is considered an important part of innate immunity owing to its bacteriostatic and bactericidal activities (Ellison and Giehl, 1991; Farnaud and Evans, 2003). The iron depleted form of lactoferrin, apolactoferrin, has bacteriostatic activity as apolactoferrin can chelate iron from its surroundings thus inhibiting many bacterial metabolic pathways (Shaper et al., 2004). Lactoferrin in the human nasopharyngeal airway binds to the pneumococcal surface protein A (PspA) preventing it from adhering to the host cells and it also degrades extracellular DNA required for the formation of the biofilm thus inhibiting colonization (Hakansson et al., 2001; Angulo-Zamudio et al., 2019). Lactoferrin is also known to have bactericidal effects against planktonic cocci (Angulo-Zamudio et al., 2019). The glycans on lactoferrin are critical for the function of this important immune effector protein. Shaper et al. demonstrated that the ability of lactoferrin to bind to the pneumococcal PspA decreased significantly upon treatment with purified *Clostridium perfringens* neuraminidase (Shaper et al., 2004). It was further demonstrated that glycosylation levels of purified lactoferrin protein decreased upon treatment with wild type D39 *S. pneumoniae* expressing NanA while no significant difference in glycosylation was observed when lactoferrin was incubated with a *nanA* deletion mutant strain (King et al., 2004). These observations suggest that NanA obstructs the lactoferrin mediated killing of the *S. pneumoniae* thus protecting the pathogen from yet another form of host immune activity.

### Modification of secretory immunoglobulins

Immunoglobulin A (IgA) is the most abundant class of immunoglobulin produced against an antigen/pathogen and plays an important role in immune homeostasis at the mucosal surfaces such as the upper and lower airway and gut by immune exclusion (Marchenko, 1982; Corthesy, 2013). IgA is present in three distinct structural forms, monomeric (mIgA), polymeric (pIgA), and secretory (sIgA). The IgAs against the polysaccharide capsule as well as other surface antigens such as PspA play an important role in limiting *S. pneumoniae* infections (Janoff et al., 1999; Kim et al., 1999; Fasching et al., 2007; Fukuyama et al., 2010). Several studies have shown that capsular polysaccharide specific sIgA as well as pIgA facilitate complement mediated phagocytosis of pneumococci (Janoff et al., 1999; Kim et al., 1999; Fasching et al., 2007). The binding of serotype specific sIgA to the pneumococcal surface activates C2-independent alternate complement pathway eventually leading to the killing of the bacteria by phagocytes (Janoff et al., 1999). It is to be noted that there are two IgA subclasses, IgA1 and IgA2. The predominant form IgA1 constitutes up to 90% of total mucosal IgA whereas IgA2 makes up for the rest of the IgA (Kett et al., 1986). The pneumococcal surface associate IgA1 protease cleaves sIgA1 at the hinge region interfering with IgA1 mediated opsonophagocytosis (Fasching et al., 2007; Janoff et al., 2014). However, IgA2 is not cleaved by IgA1 protease and could still promote complement mediated phagocytosis *in vitro* as well *in vivo* (Janoff et al., 2014). IgA2 has several sialylated glycans. King et al. demonstrated that NanA removes the sialic acid residues from IgA2 glycans and impairs its functioning. Due to the changes in the IgA molecular structure post desialylation, it can no longer bind to the bacteria to initiate antibody dependent immune response (King et al., 2004).

### Impairment of inflammatory response and neutrophil recruitment?

**Figure 2:**
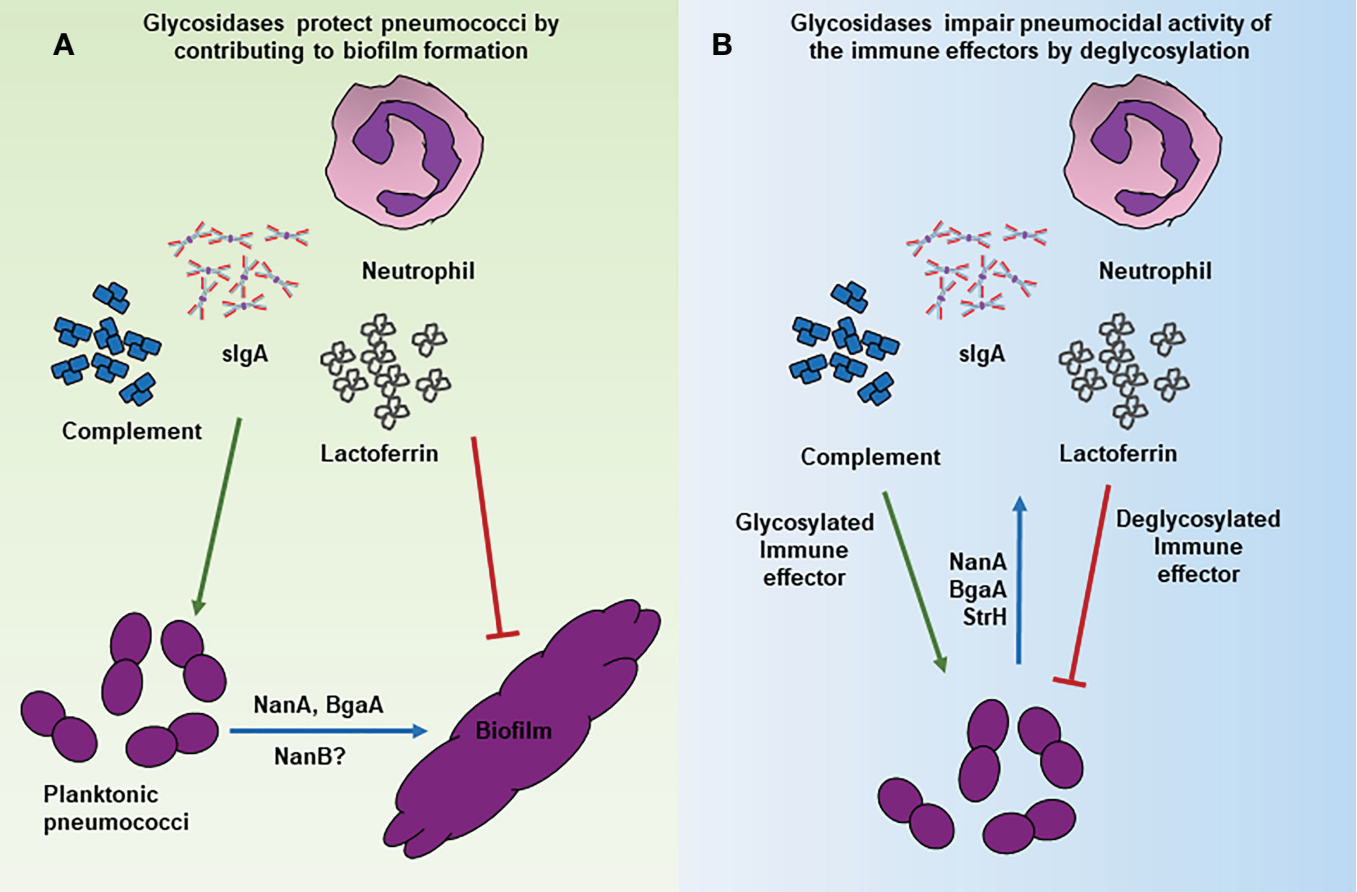
Mechanisms of glycosidase mediated immune evasion by *S. pneumoniae.* Schematic representation of various mechanisms through which surface associated glycosidases contribute to immune evasion. **(A)** NanA and BgaA contribute to the formation of biofilms that are resistant to killing by the complement mediate opsonophagocytosis/phagocytosis. **(B)** Deglycosylation of the immune effectors such as lactoferrin, IgA2 and complement proteins by NanA and other glycosidases decreases their pneumocidal activities likely by affecting their stability and/or interaction with target molecules.

It is apparent from several studies that the pneumococcal surface glycosidases can deglycosylate a variety of the host glycoproteins with the peptide chain having little to no effect on the activities of these enzymes. This begs the question if these enzymes can deglycosylate immune receptor(s) on the host cell surface and thereby affect their biological function? Tumor Necrosis Factor alpha (TNFα) and Iinterleukin-1 (IL-1) signaling is critical for the activation of robust inflammatory responses and recruitment of neutrophils during pneumococcal infection of the airway (Jones et al., 2005; Kafka et al., 2008). Mice deficient in TNFα and IL-1 receptors (IL-1R) were shown to be more susceptible to pneumococcal infection and had a high bacterial load in the lungs (Jones et al., 2005). The role of IL-1 signaling in containing the pneumococcal infection was highlighted by the observation that mice deficient in IL-1β had a significantly higher bacterial load in the nasopharynx and lungs (Kafka et al., 2008). Glycosylation of IL-1R1 is critical for its ligand binding ability, and therefore, subsequent signaling. Removal of N-linked glycans on IL-1R1 by N-glycanase resulted in a 100-fold decrease in its binding affinity to IL-1β *in vitro* (Einstein et al., 1996). Considering that the pneumococcal exoglycosidases, NanA, BgaA, StrH, and EndoD can remove glycans from a variety of host cell surface glycoproteins, we propose that pneumococcus deglycosylates IL-1R1 to avoid IL-1β mediated responses including the production of inflammatory cytokines IL-6, IL-8 and the recruitment of macrophages to the site of infection (**Figure 2**).

## Conclusion

Despite the introduction of vaccines, *S. pneumoniae* remains a major human pathogen, especially in children and elderly populations. Several virulence factors contribute to the successful colonization pathogenesis of this seemingly harmless commensal. Pneumococcal glycosidases contribute to the multiple aspects of pneumococcal biology, beginning from the colonization of the organism in the nasopharynx to providing protection against host mediated immunity during disease state. The majority of the influence of these enzymes on pneumococcal persistence and virulence could be attributed to their role in adherence, biofilm formation, nutrient acquisition, and modulation of the host immune effectors (**Figure 2**). The versatile role of these enzymes in pneumococcal biology makes them attractive targets for therapeutics. However, caution should be taken as pneumococcus is a genetically diverse species and many of the functions of these enzymes are strain specific and observation made with one strain may not be recapitulated in other strains.

## Author contributions

AS conceived the idea of the manuscript, AS, BM, PG surveyed the literature and wrote the manuscript, TN, RR, ShG, SC, SdG, SP, DB, and AV reviewed and provided critical inputs. All authors contributed to the article and approved the submitted version.
